# Assessing adverse childhood experiences in young refugees: a systematic review of available questionnaires

**DOI:** 10.1007/s00787-023-02367-6

**Published:** 2024-03-07

**Authors:** Shaymaa Abdelhamid, Eline Kraaijenvanger, Joachim Fischer, Maria Steinisch

**Affiliations:** 1 Medical Faculty Mannheim, Center for Preventive Medicine and Digital Health (CPD), Division of General Medicine, Heidelberg University, Alte Brauerei, Röngtenstraße 7, 68167 Mannheim, Germany; 2 Department of Child and Adolescent Psychiatry and Psychotherapy, Central Institute of Mental Health, Heidelberg University, J5, 68159 Mannheim, Germany; 3 Medical Faculty Mannheim, Center for Preventive Medicine and Digital Health (CPD), Division of Public Health, Social and Preventive Medicine, Heidelberg University, Alte Brauerei, Röngtenstraße 7, 68167 Mannheim, Germany

**Keywords:** Adverse childhood experiences (ACEs), Refugee children, Systematic review, Questionnaires

## Abstract

Today, various questionnaires are available to assess Adverse Childhood Experiences (ACEs) in children; however, it is uncertain if these questionnaires are comprehensive in addressing adversities of vulnerable subgroups, specifically refugee children. This review’s objectives are to (1) identify current ACE questionnaires and determine if they are suitable in assessing refugee children’s adversities, and (2) identify those previously used within a refugee population. A systematic literature search was conducted across five databases for articles published since 2010, including studies using an ACE-questionnaire that recognized multiple adversities in healthy children and were published in English. A total of 103 ACE questionnaires were identified in 506 studies. Only 14 of the 103 questionnaires addressed a refugee-specific adversity. Their ability to capture refugee children’s experiences was limited: available questionnaires used a maximum of three items to assess refugee-specific adversities, covering only a fraction of forms of adversities relevant to refugee children. Psychometric characteristics were rarely reported. In addition, only two ACE questionnaires were used within a refugee population. With the tools currently available, it is not possible to comprehensively assess the exposure to and severity of the adversities faced by refugee children. The perpetuation of ongoing crises necessitates assessing refugee children’s adversities to understand how their wellbeing is affected and to identify children at risk.

**Supplementary Information:**

The online version contains supplementary material available at 10.1007/s00787-023-02367-6.

## Introduction

Adverse Childhood Experiences (ACEs) are highly stressful and potentially traumatic events or situations that occur during childhood and/or adolescence [1]. The Centers for Disease Control and Prevention (CDC) and the Kaiser Permanente popularised this term in their pioneering study exploring the effects of adversity on people’s health and behaviour [2]. Their research revealed that children who experience an increasing number of adverse events are more likely to face serious life-long consequences in adulthood such as alcohol abuse and have increased odds for non-communicable diseases such as diabetes or chronic obstructive pulmonary disease [2]. The last two decades of research revealed that ACEs are common and rarely occur individually; thus, they have become of global importance [3].

Given global estimates that over half of all children between ages 2 and 17 (i.e., over one billion children) experienced some form of adverse event [4], a variety of tools for screening and assessing ACEs in children and adolescents have been developed. These tools aim to identify children most at risk, encourage prevention of further exposure to ACEs, help determine an appropriate treatment for children who had been exposed as well as drive policy and action to better tailor health care measures based on an understanding of how many children might suffer [5].

Some questionnaires primarily focused on a specific type of event (e.g., child sexual abuse), others examined several ACEs yet are limited to the perpetrator (e.g., family member or caregiver) or location (e.g., within the home or at school) [6]. The conventional ACEs (originating from the CDC-Kaiser Permanente study) concentrated on adversities within the home: physical, emotional, and sexual abuse, physical and emotional neglect, and household dysfunction [7, 8]. However, given that a child’s wellbeing can be affected by the community and society in which they live, it is also important to acknowledge experiences in these settings that contribute to a child’s quality of life [9]. More recent work therefore includes experiences referred to as expanded ACEs that assess exposures, such as crime, discrimination, poverty, parents’ unemployment, food insecurity, and bullying [10, 11]. As the recognition of various potential adversities within the general population develops, assessing the adversities of minority populations should also be prioritised. To some extent, vulnerable subgroups of children benefit from questionnaires designed for assessing both conventional and expanded ACEs, but it remains uncertain whether these existing tools adequately capture specific ACEs that could be more prevalent among certain high-risk child subgroups. Emphasizing the importance of assessing adversities of minority populations is crucial—those hardships, often overlooked, may significantly influence the health of vulnerable children. By addressing these adversities, researchers can advance their understanding of the underlying causes of healthcare disparities [12]. Despite the expansion of types of adversities, it is unclear whether existing questionnaires are extensive enough to encompass ACEs that may occur in subgroups of vulnerable children.

Refugee children represent one such subgroup given their frequent exposure to adverse experiences [12], including escaping from war zones, violence, conflict or persecution to find safety in another country—often without warning [13]. The estimated number of forcibly displaced children in 2021 was 36.5 million [14] with more than 4 million children resettling abroad or being internally displaced in 2022 as a result of the war in Ukraine [15]. As nearly 600 million young people live in conflict-prone regions, it is likely these numbers will be sustained or even increase in the future [16].

The adverse events encountered during flight appear to have profound effects, especially in children. Developmental and epidemiological studies suggest, for example, that displacement, detention, separation from family, and resettlement [17] may have long-lasting physical and psychological consequences [18], including a higher prevalence of post-traumatic stress disorder (PTSD), anxiety, depression, and conduct disorders [19].

The utility of available ACE questionnaires in assessing these refugee-specific ACEs is currently unclear. The purpose of this review is to identify available questionnaires that assess ACEs in children, and (1) to examine whether and to what extent these questionnaires may be useful in assessing the diverse and often unique adverse experiences encountered by refugee children and (2) to examine which ACE questionnaires have already been used within a refugee population. Identifying gaps in current ACE questionnaires may help guide the development of tools for children subjected to the refugee experience.

## Methods

### Search strategy

A search of articles published since January 2010 was conducted in four databases: PubMed, Web of Science Core Collection, PsychINFO, and Academic Search Complete. The systematic literature search was initially conducted on October 9, 2018, with updates on February 14, 2020, and on March 1, 2022. The latter two updates were made to identify publications capturing more recent refugee events. The search terms included abuse, sexual abuse, neglect, maltreatment, trauma, violence, stress, household dysfunction, adverse child experience, adverse childhood event, child, infant, adolescent, teenager, youth, questionnaire, and survey. These terms were searched for not only in the titles, abstracts, and metadata but also in the full texts of the articles, employing the ‘text word’ function. Controlled vocabulary thesauri were also employed depending on the database. The full search strategy for all databases can be found in the supplementary material. To avoid limiting the search and broaden the scope of findings, the term “refugee” was intentionally omitted from the search criteria. The rationale for this approach was to cast a wider net to capture any potential questionnaires, that might pertain to refugee experiences, regardless of the target group of the identified questionnaire. Within the broader ACE-questionnaire literature some questionnaires might be relevant to refugee children, even if not explicitly intended for the application in refugee populations. During the screening process, identified studies were meticulously reviewed to determine whether refugees (including asylum seekers and displaced people) were part of the study population. The study protocol was registered on the international database of prospectively registered systematic reviews PROSPERO (ID: CRD42019121587).

This review aims at identifying questionnaires that recognize multiple adversities in children, as previous literature has identified that many children experience multiple co-occurring ACEs that might impact their wellbeing [20, 21]. Thus, included articles used questionnaires that measured more than one ACE, in children under the age of 18 (as per the United Nations definition of a child) [22]. The included articles were published in English [23]; however, the language of the questionnaire was not constrained. As interest was only in questionnaires, studies were excluded if adversities were assessed using interviews in the form of a structured conversation. In addition, unspecified questionnaires were excluded, which entailed the omission of articles that lacked clarity regarding the specific items utilized to assess ACEs. Similarly, articles that incorporated questions from various questionnaires were also subject to exclusion in the review. This is because the analytical unit of this review was entire questionnaires, rather than individual items or questions. Our aim was to establish transparency and methodological consistency; we thus concentrated on complete questionnaires, which employed a standardized and well documented evaluation of ACEs that could be consistently applied across various research projects.

Exclusion criteria encompassed articles evaluating children with pre-existing mental disorders. Given the potential variations in how mental disorders can be measured and defined, their inclusion was avoided to maintain the focus on children from general population settings. Furthermore, the choice to emphasize the general population aligns with the World Health Organization’s priority of obtaining population-wide prevalence rates for childhood adversity through representative surveys conducted outside of clinical contexts [24]. Our intention was to ensure that the surveys we identified detect children who may require assistance, rather than specifying the reasons why children with mental health problems are seeking clinical care. Additionally, owing to inherent differences between adults and children such as limited vocabulary, cognition, experiences, and understanding [25], studies that used the same questionnaire to measure ACE exposure in both adults and children were also excluded, as the questionnaire was not designed specifically for children.

### Screening

**Fig. 1:**
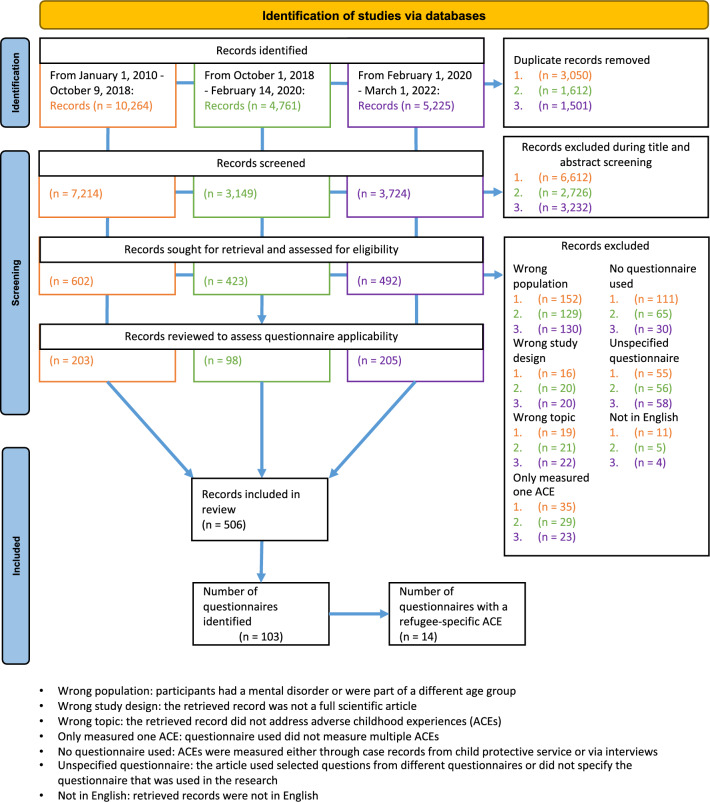
Method of identifying articles and questionnaires (adapted from the PRISMA flow diagram [26])

Two reviewers independently screened all identified articles at the title, abstract, and full-text level using Rayyan (a free web application developed by Qatar Computing Research Institute) to facilitate the screening process. Figure 1 displays the process of selecting articles included in this study. Disputes over the eligibility of studies were resolved through discussion until consensus was achieved.

### Data extraction and item assessment

A standardized data extraction form, developed by the first author, was used when reviewing information found in a study reporting use of a questionnaire. Extracted data included the country where each study took place, study population characteristics, mode of data collection, name of the questionnaire, questionnaire items, and psychometric properties.

Questionnaire items were retrieved either from the published article, survey websites, or from personal communication with the original study authors. In this review, ACEs were categorized into 11 categories defined by the research team. The first six categories as displayed in Table 1 were referred to as the conventional ACEs originating from the CDC-Kaiser Permanente study [7, 8]. The following four categories were considered expanded ACEs including adversities identified in recent literature such as community violence [27–31]. ACEs were classified as refugee-specific based on the definition of a refugee and also guided by our recent qualitative findings [32]. A refugee is someone who has been forced to flee his or her home because of persecution, war, or violence [13]; accordingly, refugee-specific ACEs include, but are not limited to, exposure to war/conflict, shootings, bombs and riots, displacement, and family separation. Different forms of adversities for each category are listed in Table 1 (expanded on findings by Laurin et al. [33]).

**Table 1:** ACE categories and forms of adversities

ACE category	Forms of adversities
**Conventional ACEs**	
Emotional abuse	A child’s family member: Verbal abuse: swore, insulted or put them down Threatening: behaved in a way that made the child fearful they would be physically harmed Inadequate nurturing: said things such as not wanting the child or wished the child were dead Tormenting: afflicted mental suffering by hurting the child’s pet, withholding a meal, or singling out the child to do chores
Physical abuse	A child’s family member: Bodily harm: pushed, grabbed, slapped, etc. the child Use of hard object/weapon: hit child with a belt, cord, etc. or cut child with sharp object Punishment: employed harsh treatment as a retribution for an offence such as wash mouth with soap or pepper, made the child dig, slash a field, or other labour as punishment Confinement: tied the child up, gagged the child, blindfolded them, or locked them in a closet or a dark place
Emotional neglect	Affectional needs not met: child often felt unimportant, unloved, unsupported and/or unprotected
Physical neglect	The failure, refusal or inability on the part of a caregiver (for reasons other than poverty) to provide for their child’s: Material needs: child sometimes went without food, clothing, shelter or protection Medical needs: child was not taken to the doctor when sick Supervisory needs: parents did not ensure a safe place for child to stay, child left at home alone, or child was left in charge of younger siblings for long periods of time
Sexual abuse	Physical sexual abuse: someone attempted to have sexual intercourse with the child, touched the child’s private parts, or asked child to touch their private parts in a sexual way that was unwanted, uncomfortable or against child’s will Verbal sexual abuse: someone said/wrote something sexual about the child, talked to child in a sexual way or made sexual comments about child’s body Unwanted sexual exposure: someone attempted or made child watch sexual things (e.g., magazines, pictures, videos, internet sites), made child look at their private parts or wanted child to look at theirs, took sexual picture/video of child, or child was present when someone was being forced to engage in sexual activity Threatening: someone threatened to have sex with child, or hurt/tell lies about them unless they did something sexual Transactional: child traded sex or sexual activity to receive money, food, drugs, alcohol, a place to stay, or anything else
Family dysfunction	Parental separation or divorce: child’s parents were divorced or separated Domestic violence: child witnessed a parent hit, slap, kick, push or physically hurt another parent or siblings, child has seen or heard family members arguing very loudly or threaten to seriously harm each other Mental illness: a family member was depressed, mentally ill, or (attempted) suicide Substance abuse: a family member was a problem drinker/alcoholic or used street drugs Incarceration: a family member served time in jail or was taken away (by police, soldiers, or other authorities)
**Expanded ACEs**	
Community violence	Interpersonal violence committed in public areas by individuals who are not intimately related to the child. Examples include: Crime: robbery, theft, vandalism, exposure to drug activity Assault: child witnessed or was exposed to being attacked with/without an object or weapon Kidnapping: child was kidnapped Discrimination: child was hit or attacked verbally because of skin colour, religion, family origin, physical condition, or sexual orientation Killing: heard about or witnessed murder Use of a weapon: heard about or witnessed random shootings/stabbings
Economic hardship	Child’s family facing financial hardship: Financial instability: income loss, unemployment, job instability, was not able to afford food and necessities Housing insecurity: child was living in a car, a homeless shelter, a battered women’s shelter, or on the street
School victimisations	Physical violence: another child and/or teacher physically hit, kicked, pushed, or took things forcibly from the child Psychological stressors: another child and/or teacher emotionally mistreated a child by social exclusion, threatening relationship termination, gossip and secret spreading Sexual offence: another child or teen pressured the child to do sexual things or did something sexual to child against their wishes Bullying: child threatened or harassed by a bully Online victimisations: cyber bullying or online sexual harassment
Other	Dating violence: was hit, verbally hurt or controlled by partner Accident: experienced/witnessed a serious car/bicycle accident, near drowning experience or fire Natural disaster: child experienced a disaster such as a tornado, hurricane, big earthquake, flood or mudslide Severe illness/Medical trauma: child or loved one had to undergo frightening medical treatment or was hospitalized for a long time period Animal attack: child was badly hurt by an animal Bereavement: death of someone close to the child Familial changes: child was completely separated from parent/caregiver for a long time under very stressful circumstances, such as going to a foster home, the parent living far apart from them, or never seeing the parent again. Addition of third adult to family (e.g., marriage of parent to step-parent) Child detention: child was detained, arrested or incarcerated Difficulties: the child moved to a new school, home, or town, repeated a grade in school, etc.
**Refugee-specific ACEs**	
Refugee-specific adversities	War/conflict: child was exposed to war or conflict Shootings, bombs, and riots: child saw or heard people being shot, bombs going off, or street riots Displacement: child was forced to flee their home Beaten up by soldiers, police, militia, or gangs: child was hurt badly by armed adults Family separation: child was separated from their caregiver due to immigration or war

Psychometric properties help assess the degree to which a questionnaire measures the desired content and whether the data it yields are reproducible [34]. Therefore, as a quality assessment, we identified whether retest reliability, internal consistency, inter-observer reliability, content validity, criterion validity, construct validity including cross-cultural validity (if applicable) had been reported as outlined in previous work by de Souza et al. [34].

A second research team member independently crosschecked all the extracted data of 100 randomly selected articles with the original articles. Minor differences, such as labelling of demographic information, were discussed until agreement was achieved. Since there were no major differences, duplicate checking of all reports was deemed unnecessary.

### Analytic strategy

The unit of analysis in this study is the questionnaire itself. To determine whether ACE questionnaires are useful in evaluating adversities that refugee children may encounter, a descriptive record of characteristics (i.e., the adversity categories and forms measured and psychometric properties reported) was made. With attention on those questionnaires that measured refugee-specific ACEs, the analytic strategy involved two focuses: (1) to record which questionnaires measured which form of adversity in each of the ACE categories and (2) to record the number of questions addressing each category to examine the extent to which each adversity category had been measured; the use of multiple rather than single questions indicated a higher extent.

To examine the quality of the questionnaires, information regarding three aspects of reliability and four aspects of validity was extracted from all the studies identified in this review that reported this information. For the questionnaires that addressed a refugee-specific ACE, the number of studies that reported on the individual psychometric aspects was assessed.

## Results

A total of 506 full-text articles fulfilled the inclusion criteria, in which use of 103 unique questionnaires was reported. Questionnaires were administered mostly via self-report (*n* = 67 questionnaires), and data collection often was by means of a school based survey (*n* = 45 questionnaires). The number of participants varied from 14 to 29,696,808, as some of the questionnaires were used as part of national surveys. Approximately half of the questionnaires were employed  in the United States of America (USA) (*n* = 48 questionnaire), and most of the questionnaires were in English (*n* = 57 questionnaires).

### Adversities measured

**Fig. 2:**
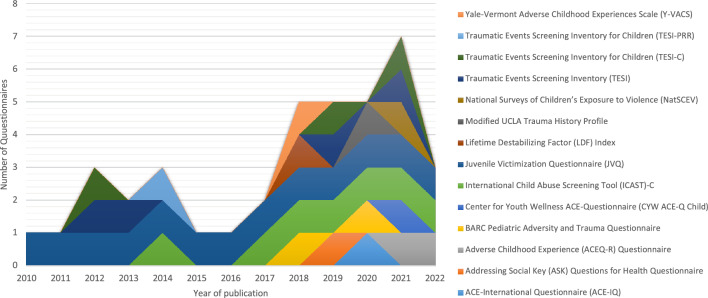
Annual distribution of the 14 questionnaires

The identified questionnaires measured different adversity categories that could be relevant to refugee children, yet only 14 included one or more questions addressing a refugee-specific adversity as listed in Table 1. Figure 2 presents a summary of these 14 questionnaires, specifying the years they were employed.

**Fig. 3:**
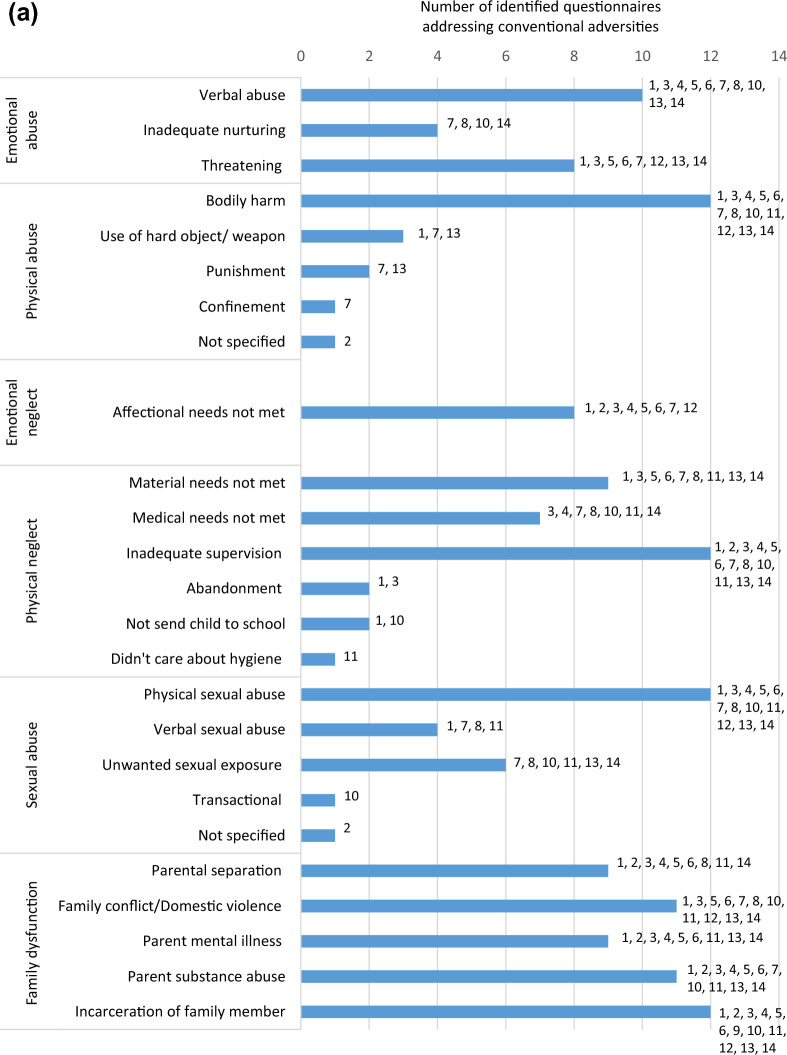

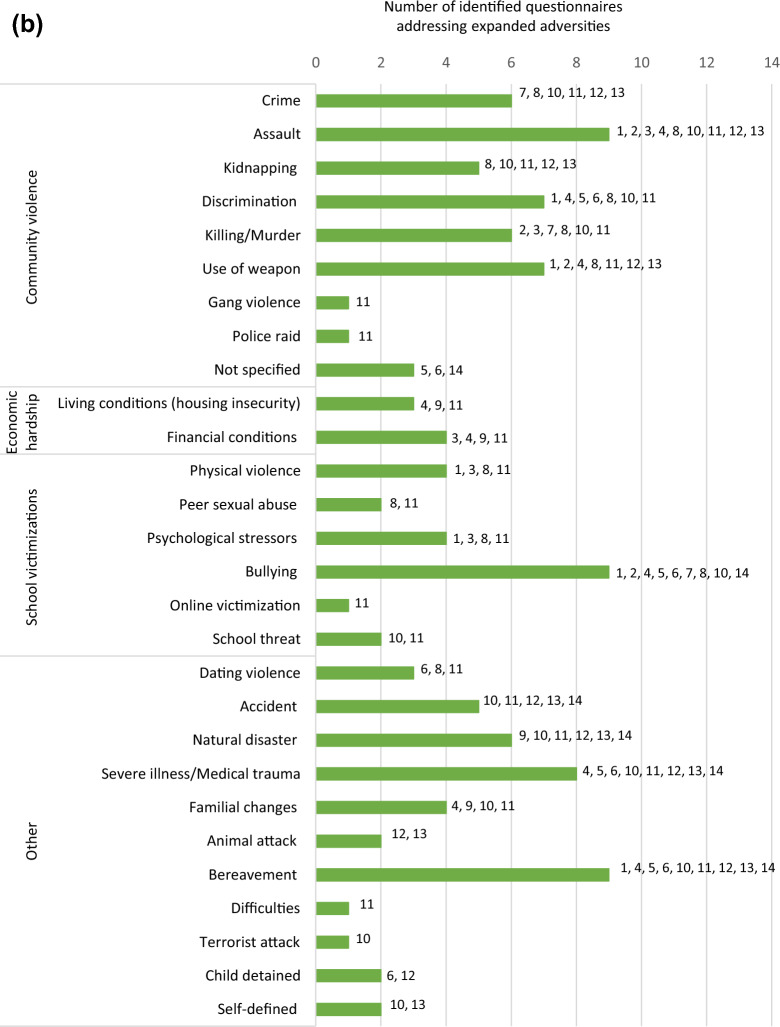

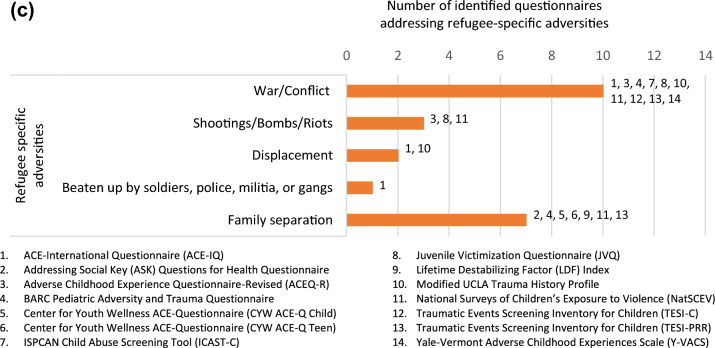
**a** Conventional ACEs addressed by identified questionnaires. **b** Expanded ACEs addressed by identified questionnaires. **c** Refugee-specific ACEs addressed by identified questionnaires

Exposure to war/conflict and family separation were the forms of refugee-specific ACEs being addressed most frequently with ten questionnaires addressing war/conflict and seven addressing family separation. Being beaten up by soldiers, police, militia, or gangs was addressed by only one questionnaire, displacement by two and exposure to shootings/bombs/riots by three questionnaires. An overview of which form of adversity is assessed by which of these 14 questionnaires can be found in Fig. 3a–c.

Within the identified 14 questionnaires, between one and three questions addressed refugee-specific adversities, whereas other categories were addressed by up to 21 questions such as community violence in the National Surveys of Children’s Exposure to Violence (NatSCEV) [35]. In the NatSCEV, questions about community violence included “Has your child ever lived in a neighborhood where there were gangs?” as well as “At any time in your child’s life, has your child ever seen the police raid or enter a house in (his/her) neighborhood looking for a criminal or block off a place in (his/her) neighborhood because a crime happened there?” Table 2 shows the number of questions addressing each adversity category in each questionnaire.

**Table 2:**
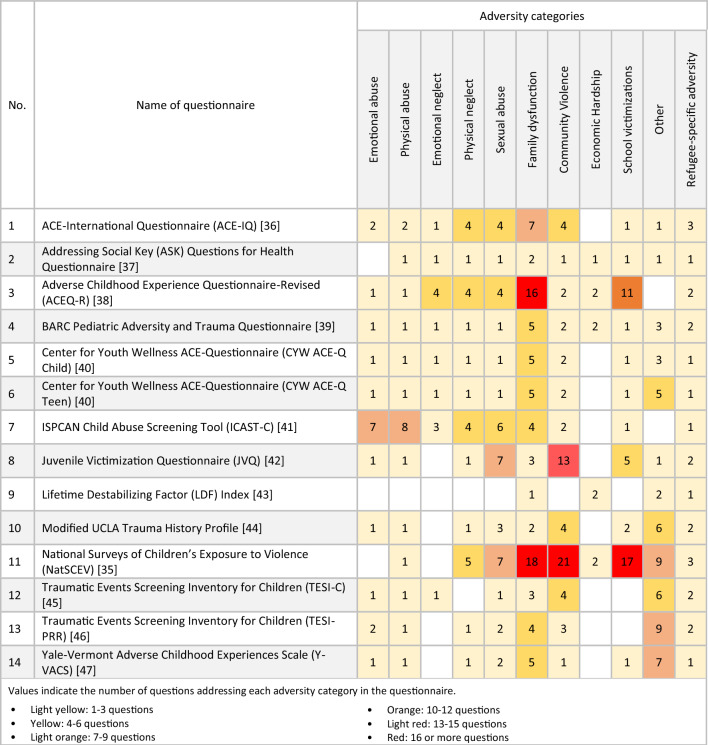
Adversity categories in identified questionnaires

### Psychometrics and questionnaire quality

Most articles did not report any information related to the psychometric properties of the questionnaire used. From the 14 questionnaires that included a refugee-specific adversity, three questionnaires (JVQ, ICAST-C, and ACEQ-R) had reported psychometric properties. In this review, these three questionnaires were used in more than one study; however, their psychometric properties were not always reported. For instance, as displayed in Table 3, the JVQ was used in 76 different studies, of which only 15 had reported internal consistency (Cronbach’s alpha or Kruder-Richardson Formula 20) and eight reported inter-observer reliability (kappa statistics). The JVQ was originally developed in the USA and was used in nine countries beyond the USA within 39 studies identified in this review; yet only four identified studies reported the translation process.

**Table 3:**
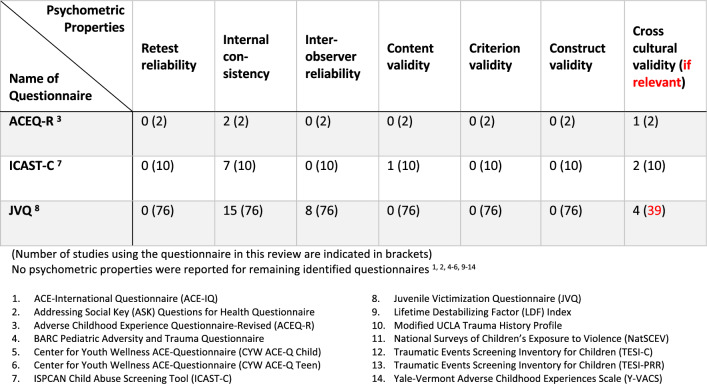
Reported psychometric properties of identified questionnaires

In the evaluation of questionnaire reliability and validity, the ACEQ-R demonstrated internal consistency with reported values of 0.77 and 0.87 in two respective articles. The ICAST-C questionnaire exhibited a wider range of internal consistency values, spanning from 0.35 to 0.96. Similarly, the JVQ displayed a diverse range of internal consistency, varying from 0.43 to 0.98. Regarding inter-observer reliability, only the JVQ had reported values within the range of 0.52 to 0.75. As for content validity, one study reported that content validity for the ICAST-C was developed through expert psychologists’ opinions. As indicated in Table 3, very few articles discussed the cross-cultural adaptation of the questionnaire used. Of those that did, some provided a good description such as “[the ACEQ-R] was first translated to Chinese, followed by back-translation. Three bilingual experts were invited to verify the translation along with 30 adolescents who engaged in a pre-test to determine its readability” [48]. Others solely stated the translation methods, such as forward translation [49] or back-translation [50], without discussing cultural adaptation. None of the studies reported retest reliability (interclass correlation coefficient value), criterion validity (correlation coefficient), or construct validity (convergent validity, discriminant validity, or confirmatory factor analysis).

### Questionnaires used within a refugee population

In this review, two questionnaires were used to measure ACEs in refugee children, the first questionnaire was the ISPCAN Child Abuse Screening Tool-Child (ICAST-C), used as a self-report measure in one study with children in refugee camps in Rwanda and Uganda [51]. The other questionnaire was the ISPCAN Child Abuse Screening Tool-Parent (ICAST-P), applied in a study asking mothers about the adversities of their Palestinian children, some of whom were displaced refugees in the West Bank [52]. While the ICAST-C addresses refugee-specific ACEs, this is not the case with the ICAST-P.

## Discussion

The purpose of this review was to determine if any of the existing questionnaires assessing ACEs also capture the diverse and unique adversities faced by refugee children and if so, to what extent. Further, we analysed which of the available ACE questionnaires had been applied in a refugee setting. This study identified 103 questionnaires that measure various adversity categories yet the core content included within the questionnaires differed, as did the questionnaire quality. Due to the high number of questionnaires identified, it has become apparent that ACEs are perceived globally as a problem. However, it seems that some vulnerable populations, such as refugee children, currently might go unrecognized.

### Adversities measured

The range of adversities covered in questionnaires available today has expanded immensely, since the conventional ACEs were first revealed in 1998 [2]. Not only do current adversities vary in source (the child’s family or environment) but also in severity and exposure (either witnessed or experienced). However, when assessing the applicability of the identified ACE questionnaires for recognizing the adversities faced by refugee children, a notable constraint emerges. Without a doubt, it is important to acknowledge that the conventional and expanded experiences like family dysfunction, abuse, or community violence are pertinent to refugee children [53–55]. Nevertheless, these are not the only pertinent adversities. Assessing refugee children’s adversities with such measures can be misinformative as has been shown in a study measuring ACEs among Latino immigrant youth and young adults (aged 10–20) in the United States (US). In this study, Latino participants completed a conventional ACE-questionnaire and a novel 13-item measure of immigrant-specific ACEs (ACE-I). ACE-I items tended to receive higher endorsement, suggesting that there are specific ACEs for Latino immigrants that were not previously considered [56]. In addition, 15% reported experiencing no conventional ACEs [56]. Such results highlight that solely using a conventional or expanded ACE measure without including refugee-specific items can mask the actual experiences of this population.

In this systematic review, only 14 of the 103 identified questionnaires included a refugee-specific ACE. Examples of refugee-specific ACEs in the identified questionnaires included experiencing war, bombings, destruction, displacement, and separation from family due to immigration. However, it is important to consider that refugee children may also face adversities similar to the general public that are relevant to their circumstances, such as economic hardship, kidnapping, bereavement, and discrimination [53, 57].

Table 4 depicts an overview of potential ACEs that might be relevant to the refugee population based on previous research [58–67], including several adversity forms applicable to both the refugee and general population. For a better overview, Table 4 also shows which adversity forms have been covered by the 14 identified questionnaires that included at least one refugee-specific item. It becomes apparent that none of the identified questionnaires addressed all forms of adversities—furthermore, several forms of adversities relevant to refugee populations (such as military groups, immigration detention, immigration stress, and acculturation stress) were not addressed within any of the questionnaires identified in this study.

**Table 4:**
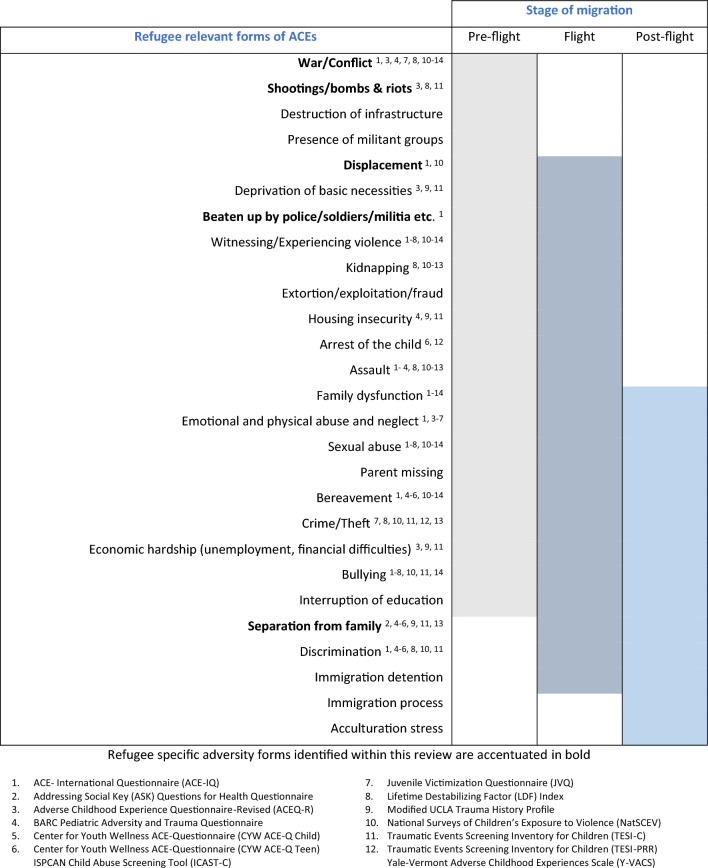
Migration stages in which refugee-relevant ACEs occurred (based on previous research [58–67])

One questionnaire,: the BARC Pediatric Adversity and Trauma Questionnaire [39], covered all ACE categories employed in this review; measuring at least one form of adversity out of each category, most of them being assessed with one or two questions (see Table 2). However, when looking at the breakdown of adversities as depicted in Table 4 it is noticeable that certain issues are missing: kidnapping (a form of community violence), a reality many refugee children might face [57], is not addressed in the BARC questionnaire. Neither is financial difficulties [68], displacement [59], or immigration process [65], all common refugee adversities acknowledged in earlier research.

Furthermore, taking a closer look at the extent to which refugee-specific ACEs are measured in the 14 identified questionnaires, it is noticeable that their measure is limited with a maximum of three refugee-specific questions. For example, the NatSCEV, only addressed refugee-specific ACEs with three questions (war/conflict, shootings/bombs/riots, and family separation), while community violence and family disfunction were addressed with 21 and 18 questions respectively, as shown in Table 2. Six of the fourteen questionnaires used two questions to ask about refugee-specific ACEs (ACEQ-R [38], BARC [39], JVQ [42], Modified UCLA [44], TESI-C [45], and TESI-PRR [46]), and another six tools used only one question (ASK [37], CYW ACE-Q Child [40], CYW ACE-Q Teen [40], ICAST-C [41], LDF [43], and Y-VACS [47]). Additionally, the majority of questionnaires addressed refugee-specific ACEs by asking about exposure to war/conflict and/or separation from family. Despite the damaging influence of such exposures, they are not the sole sources of adversity among refugee children. It is therefore unjust to simplify refugee-specific adversities into three questions or less. Simply addressing only a few forms of adversities understates the gravity of the refugee experiences, as well as their mental and physical consequences.

It was also noticeable that the questionnaires addressing refugee-specific adversities focused on pre-flight and flight stressors. The tragedies refugee children experience are not only occurring in their home countries, but also on the dangerous route to safety, and in their host countries [69]. In some circumstances, the adversities faced post-flight have caused more of a negative impact on refugee’s wellbeing than that of war and conflict [70, 71].

In addition, it is important to acknowledge that factors on multiple levels could be a source of adversity. These factors could arise from the individuals themselves, their families, community, or society—such as psychological vulnerabilities, impaired parenting, community tensions, or national policies that adversely affect refugee children. However, looking at potential factors arising from all sources with one questionnaire appears to be uncommon in ACE research.

This demonstrates that even in the 14 identified questionnaires, the refugee-specific ACEs are not capturing the full range of adversities these children may encounter. However, it has to be acknowledged that the identified questionnaires were not developed for refugee children and their importance should not be diminished in any way. It becomes apparent, nevertheless, that a gap exists concerning measuring refugee children’s adversities.

### Questionnaire quality

Most articles in this review provided little information about the psychometric properties of questionnaires used. Taking a deeper look at studies identified in this review that used a questionnaire evaluating refugee-specific ACEs, only 3 out of 14 questionnaires had psychometric properties reported (ACEQ-R [38], ICAST-C [41] and JVQ [42]), with several psychometric aspects lacking. In some cases, articles stated that the psychometric properties were reliable due to the extensive use of the questionnaire in the previous studies [72]. In other cases, vague statements such as the questionnaire has shown “acceptable psychometric properties” [73], were not sufficient to estimate the quality of the tool. The articles that did provide some details about psychometric properties mainly reported on internal consistency, as shown in Table 3. This assesses whether the items of a questionnaire measure the same characteristic, usually by providing a Cronbach’s alpha value. However, a Cronbach’s alpha value is greatly affected by the number of items in the questionnaire; by simply increasing the number of items, the alpha values are also increased; this alone is not enough to assess the questionnaire quality [34].

Additionally, there was an underreporting of cross-cultural validity of the questionnaires. In those few studies reporting on cross-cultural validity, only the translation method was described, yet the tool’s adaptability in a new cultural setting was rarely described. To minimise bias when the questionnaire is administered in a different language and context, cross-cultural adaptation is essential, and this entails both linguistic and cultural considerations [74].

While psychometric properties for the respective questionnaires might be published elsewhere not included in this review, the missing report of properties within the studies identified in this review still is problematic, because reliability and validity are subject to change according to the context, study type, population, and purpose of the study [34].

Generally, questionnaires should meet valid and reliable criteria by measuring its psychometric properties to ensure that the questionnaire measures what it proposes and reproduces the same results over time [34]. Research has also indicated that the evidence regarding psychometric properties of adversity questionnaires is limited and usually of lower quality [75, 76]. To collect accurate data, researchers should also take into account factors that influence the quality of information such as the respondent characteristics, for instance, age or cultural appropriateness [26]. Consequences of ACEs might be obscured due to the variable validity and reliability of existing questionnaires. Thus, it is imperative that the psychometric properties of questionnaires, including, if applicable, cross-cultural validity, are evaluated, to ensure that the information obtained in a study is valid and reliable and can thus be used to assist in decision-making.

### Questionnaires used within a refugee setting

Despite the magnitude of the humanitarian crisis that affects every continent [77], research to evaluate ACEs with standardized tools in refugee children is negligible. Only two questionnaires were used to measure ACEs in refugee children. The questionnaires used were the ICAST-P and ICAST-C, tools developed through a global collaborative effort guided by the International Society for the Prevention of Child Abuse & Neglect (ISPCAN). The ICAST questionnaires are ISPCAN's effort to provide a common tool for systematically comparing recorded incidences of all types of violence against children across cultures and time, to provide a more accurate and representative picture of the global problem [41]. Since the ICAST is a global endeavour it has been translated and tested in at least 20 languages [41]. In this review, the ICAST-P was used in Arabic in Palestine and the ICAST-C was used in Kinyarwanda for Rwanda and in Dinka and Nuer for Uganda.

The study which used the ICAST-P, a tool with 39 questions in total, aimed to assess the prevalence of child abuse in the West Bank, of which 30.4% of the participants were refugees, and to determine some of its associated social and political factors [52]. The ICAST-P only measures a few (emotional and physical abuse and neglect, economic hardship, and sexual abuse) of the adversity forms depicted in Table 4 and does not address a single refugee-specific adversity [41]; however, that was not the goal of the identified study.

On the other hand, the study using the ICAST-C (a questionnaire with 61 items) aimed to understand patterns of violence against children in refugee camps, and associations with adverse mental health outcomes [51]. While the ICAST-C does address a refugee-specific adversity form—exposure to war/conflict, covered by only one question—and six further adversity forms relevant to refugee children, as illustrated in Table 4, it still overlooks important struggles refugees may experience pre-flight, during flight, and post-flight, specifically discrimination and economic hardship.

It is worth noting that there exists a considerable body of research establishing a correlation between traumatic experiences in refugee populations and negative mental health outcomes. However, this existing body of research predominantly concentrates on specific forms of adverse experiences and their impact on health, such as the exposure to war and the correlation with PTSD [78]. However, the aim of this review was to identify standardized measurement tools of exposure to various forms of adversities, not studies assessing the effect of certain predefined single exposures. With a continuously growing refugee population, and previous research suggesting mental and physical health disorders related to traumatic experiences refugee children encounter [79], incorporating the adversities affecting their wellbeing into ACE questionnaires becomes highly important.

### Limitations and strengths

This is the first systematic review evaluating whether and to what extent the existing ACE questionnaires can be used to identify the adversities of refugee children. However, it is important to acknowledge a few limitations of our study.

First, studies may have employed alternative data collection methods, such as interviews, which, while valuable for qualitative research, entail open-ended responses and narratives that may not align with the analytical approach adopted for this systematic review. The deliberate choice to focus on structured questionnaires and surveys allowed us to maintain methodological consistency and effectively address our research questions. Moreover, some existing questionnaires might not have been identified, because our search was limited to articles published in English, thus limiting the international scope of the review. Additionally, articles were excluded if some respondents were outside the desired age range, because they did not fit the definition of a child, thus inferring that the questionnaire is not explicitly designed for children.

Another potential limitation is that articles with unspecified questionnaires were excluded from this study. While it is possible to view the use of selected questions from different questionnaires as a new questionnaire, we decided to classify them as “unspecified questionnaires” when the article did not explicitly specify or provide a coherent description of the questionnaire used. The primary rationale behind this decision was to focus our review on studies that used standardized ACE questionnaires. We aimed to maintain clarity and rigor in our inclusion criteria to ensure that the data extracted from included studies were directly relevant to our research objectives.

Some of the excluded unspecified questionnaires might have contained valuable questions relevant to the refugee population. However, these articles lacked transparency in specifying the items used to assess ACEs. For instance, in one article the authors mentioned creating four deprivation variables and three threat variables but did not provide the actual questions used [80]. Additionally, some articles incorporated individual questions from various questionnaires, leading to their exclusion. By adopting entire questionnaires as the analytical unit, our systematic review ensures a comprehensive and consistent assessment of ACEs across diverse studies. This approach enhances the methodological rigor of this review and also promotes research reproducibility. This review may also be subjected to publication bias, as searches outside the mentioned databases were not made. Furthermore, information about the modifications made by certain studies to the original version of questionnaires was not collected.

Despite these limitations, we were able to use explicit methodology to identify 14 questionnaires on a global level that assess at least one refugee-specific ACE in children. The results provide a detailed overview of assessed forms of adversities categorized into domains to assist future researchers in identifying useful questionnaires. Additionally, this review draws attention to the existing gaps and the need for a questionnaire that addresses the unique adversities of refugee children.

## Conclusion

This review shows that regardless of the availability of numerous questionnaires, there is no one-size-fits-all measure for every situation. It also illustrates that there is a need for further psychometric development for most measures. However, the most important finding is that that there is a need to incorporate adversity measures for the most vulnerable populations, specifically refugee children. Existing questionnaires are limited in terms of the extent they address refugee adversities. Given the importance and seriousness of ongoing crises that result in the displacement of children, inclusion of adversities relevant to refugees will allow for further understanding of how ACEs affect these children’s wellbeing and enable the identification of those at risk.

## Supplementary Information

Below is the link to the electronic supplementary material.

Supplementary file1 (DOCX 20 KB)

## Data Availability

Data extracted from identified records or questionnaires supporting the findings of this study are available from the corresponding author (SA) on request.
