# Supplementary material for: Assessing adverse childhood experiences in young refugees: a systematic review of available questionnaires
Source: Eur Child Adolesc Psychiatry. 2024 Mar 7;33(12):4043–59. doi: 10.1007/s00787-023-02367-6 (PMC11618322; doi:10.1007/s00787-023-02367-6)
Supplement: Supplementary file 1 — Supplementary file1 (DOCX 20 KB) [file 787_2023_2367_MOESM1_ESM.docx]

### Supplementary material to

Abdelhamid S, Kraaijenvanger E, Fischer J, Steinisch M (2024) Assessing adverse childhood experiences in young refugees: a systematic review of available questionnaires. European Child & Adolescent Psychiatry. https://doi.org/10.1007/s00787-023-02367-6

### Supplementary File 1: Search strategy

**PubMed**

| **"child abuse"[MH] OR**  **"child abuse, sexual"[MH] OR**  Adverse Childhood Experience*[tw] OR  Adverse Childhood event*[tw] OR  Adverse child experience*[tw] OR  Adverse experience*[tw] OR  **"**household dysfunction**"**[tw] OR  "child abuse" OR  "childhood abuse" OR  "child neglect"[tw] OR  "childhood neglect"[tw] OR  "child maltreatment"[tw] OR  "childhood maltreatment"[tw] OR  "child trauma"[tw] OR  "childhood trauma"[tw] OR  "childhood stress"[tw] OR  "childhood violence"[tw] OR  "child violence"[tw] |
| --- |

| **child[MH] OR**  **infant[MH] OR**  **adolescent[MH] OR**  child*[tw] OR  infant[tw] OR  infants[tw] OR  adolescent[tw] OR  adolescents[tw] OR  baby[tw] OR  babies[tw] OR  toddler[tw] OR  toddlers[tw] OR  teenager[tw] OR  teenagers[tw] OR  teen[tw] OR  teens[tw] OR  preteen[tw] OR  preteens[tw] OR  youth[tw] |
| --- |

| Questionnaire*[tw] OR  survey*[tw] |
| --- |

**Bemerkungen**

**Filter age:** NOT ("adult"[Mesh] NOT ("infant"[Mesh] OR "child"[Mesh] OR "adolescent"[Mesh]))

**Web of Science Core Collection**

| "Adverse Childhood Experience*" OR  "Adverse Childhood event*" OR  "Adverse child experience*" OR  "Adverse experience*" OR  "household dysfunction" OR  "child abuse" OR  "childhood abuse" OR  "child neglect" OR  "childhood neglect" OR  "child maltreatment" OR  "childhood maltreatment" OR  "child trauma" OR  "childhood trauma" OR  "childhood stress" OR  "childhood violence" OR  "child violence" |
| --- |

| "child*" OR  "infant" OR  "infants" OR  "adolescent" OR  "adolescents" OR  "baby" OR  "babies" OR  "toddler" OR  "toddlers" OR  "teenager" OR  "teenagers" OR  "teen" OR  "teens" OR  "preteen" OR  "preteens" OR  "youth" |
| --- |

| "Questionnaire*" OR  "survey*" |
| --- |

**Bemerkungen**

Filter: Age Groups: Childhood (birth-12 yrs), Adolescence (13-17 yrs)

Recherche mit der Einstellung Title. Eingrenzung Publikationen ab 2008

**PsychInfo**

| **DE "Child Abuse" OR**  "Adverse Childhood Experience*" OR  "Adverse Childhood event*" OR  "Adverse child experience*" OR  "Adverse experience*" OR  "household dysfunction" OR  "child abuse" OR  "childhood abuse" OR  "child neglect" OR  "childhood neglect" OR  "child maltreatment" OR  "childhood maltreatment" OR  "child trauma" OR  "childhood trauma" OR  "childhood stress" OR  "childhood violence" OR  "child violence" |
| --- |

| "child*" OR  "infant" OR  "infants" OR  "adolescent" OR  "adolescents" OR  "baby" OR  "babies" OR  "toddler" OR  "toddlers" OR  "teenager" OR  "teenagers" OR  "teen" OR  "teens" OR  "preteen" OR  "preteens" OR  "youth" |
| --- |

| "Questionnaire*" OR  "survey*" |
| --- |

**Academic Search Complete (published first as Premier) (via EBSCO host)**

| "Adverse Childhood Experience*" OR  "Adverse Childhood event*" OR  "Adverse child experience*" OR  "Adverse experience*" OR  "household dysfunction" OR  "child abuse" OR  "childhood abuse" OR  "child neglect" OR  "childhood neglect" OR  "child maltreatment" OR  "childhood maltreatment" OR  "child trauma" OR  "childhood trauma" OR  "childhood stress" OR  "childhood violence" OR  "child violence" |
| --- |

| "child*" OR  "infant" OR  "infants" OR  "adolescent" OR  "adolescents" OR  "baby" OR  "babies" OR  "toddler" OR  "toddlers" OR  "teenager" OR  "teenagers" OR  "teen" OR  "teens" OR  "preteen" OR  "preteens" OR  "youth" |
| --- |

| "Questionnaire*" OR  "survey*" |
| --- |
